# Intending to avoid the treatment burdens only: the doctrine of double effect and withholding or withdrawing life-sustaining treatment

**DOI:** 10.1007/s11017-025-09712-7

**Published:** 2025-03-21

**Authors:** Hitoshi Arima

**Affiliations:** Graduate School of Urban Social and Cultural Studies, Yokohama City University, Kanazawa-Ku, Seto 22-2, Yokohama, Japan

**Keywords:** Doctrine of double effect, Euthanasia, Intention, Withholding or withdrawing life-sustaining treatment

## Abstract

It is often believed that withholding or withdrawing life-sustaining treatment is justifiable only when the patient’s death is not intended. Also, in accordance with this belief, many argue that the justification of withholding/withdrawing life-sustaining treatment is an application of the doctrine of double effect (hereafter DDE). This paper aims to defend these accounts from some important criticisms. Baruch Brody maintains that most people intend the patient’s death when they withhold/withdraw such treatments and that therefore, there are many cases of withholding/withdrawing treatment that are clearly justifiable but rendered unjustifiable by the accounts. Daniel P. Sulmasy asserts that withholding/withdrawing treatment rarely satisfies DDE’s fourth condition (that the good effect of the act is proportionately greater than its bad effect) because the goodness of avoiding treatment burden seldom compares to the badness of shortening life. I examine these claims and show that they are mistaken. Central to the discussion in this paper is the idea that those who withhold/withdraw life-sustaining treatment often only intend to avoid the burdens posed by the treatment itself and not to shorten the patient’s life. It will be argued that both Brody and Sulmasy are led to an erroneous conclusion because they fail to have an accurate understanding of this idea and its implications.

## Introduction

When is it morally justifiable to withhold or withdraw treatment that is necessary to sustain a patient's life? Many believe that it is only when the patient's death is not intended [1, 2, 3, pp. 18–19]. Also, in accordance with this belief, it is often understood that the justification of withholding/withdrawing treatment^1^ is an application of the doctrine of double effect (hereafter DDE) [4, pp. 417–418; 5, pp. 159–167; 6; 7, pp. 424–426; 8, pp. 71–80; 9, pp. 162–163; 10; 11, pp. 62–69]. This paper aims to defend these claims from a few important criticisms.

In general, DDE applies to an act that has at least one good and one bad foreseeable consequence. According to the doctrine’s traditional formulation, an act must satisfy all the following four conditions simultaneously for it to be justifiable [12, 13, p. 26]:

1. The act in itself is good or at least morally indifferent.

2. The agent intends the good effect and not the bad effect.

3. The good effect is not produced or caused by means of the bad effect.

4. The good effect is proportionately serious enough to permit the bad effect.^2^

The “bad effect” here refers to a consequence that one should never intend to bring about. The view that there are some states of affairs that one should never intend to bring about is called moral absolutism. Traditionally, the death of an innocent person has been understood by the doctrine’s proponents to be one of the things that should never be intended to bring about [12; 13, Ch. 1; 14, p. 58; 15, p. 476; 16, p. 465].^3^ This paper does not dispute absolutism or the claim that each of the above four is a necessary condition for an act to be justifiable.^4^ Assuming that they are correct, I will explore how they apply to withholding/withdrawing treatment. (But I will also come back to absolutism in the third section).

As to when withholding/withdrawing treatment can be justified by DDE, prior studies have made the following general statements.^5^ Withholding/withdrawing life-sustaining treatment can have two effects: shortening the patient's life (*a*) and sparing the patient from burdens imposed by the treatment (*b*), where *a* is a bad effect, and *b* is a good effect. DDE states that withholding/withdrawing a treatment is justifiable when the agent, i.e., physician, patient, and/or family, intends only *b* but not *a* (otherwise the case would violate the second of the four conditions of DDE seen above), when *a* is not the means to cause *b* (otherwise the third condition would be violated), and when *b* is good enough to make up for the badness of *a* (otherwise the fourth condition would be violated). DDE prohibits withholding/withdrawing treatment if any one of these requirements is not satisfied.^6^ Note that it is customary among these researchers to call a treatment “extraordinary” when withholding/withdrawing it is justifiable by DDE (or when it is possible to understand that the agent does not intend *a* when withholding/withdrawing it) and “ordinary” when it is not justifiable by DDE (or when it is not possible to understand that the agent does not intend *a*) [1, p. 124; 2, p. 135; 5, p. 161; 7, pp. 424–425; 9, p. 162].

However, prior studies also contain some important criticisms of these accounts. Baruch Brody rejects the view that withholding/withdrawing life-sustaining treatment is justified, when it is justified, because the death of the patient is not intended. As Brody understands, it is rarely the case that those who refuse such treatments only intend to avoid the treatment burden and not to shorten the patient's life. Thus, he argues, the view is mistaken because there are many cases of withholding/withdrawing treatment that are clearly justifiable but rendered unjustified under the view [25].

Daniel P. Sulmasy also believes that withholding/withdrawing life-sustaining treatment is justifiable in many cases but denies that DDE provides rationale for the justification. He denies this because he contends that the goodness of avoiding the burden of treatment is seldom great enough to allow for the badness of shortening life [26–28]. Additionally, Sulmasy also draws attention to the case of amputation. As he argues, in amputation, the bad outcome of removing a limb is the means to cause the good outcome of bringing health; hence, DDE leads to the obviously erroneous conclusion that amputation must always be foregone [29]. (Sulmasy also opposes equating extraordinary treatments and treatments that can be justifiably withheld/withdrawn under DDE [27]. I will also discuss this later.)

The purpose of this paper is to refute these claims. Central to the discussion in this paper is the idea that those who forego life-sustaining treatment often intend only to avoid the burdens imposed by the treatment itself but not to shorten the patient’s life. I will argue that both Brody and Sulmasy fail to have a correct understanding of this idea or its implications. My argument will also clarify some of the key steps that need to be followed when applying DDE to the withholding/withdrawing of treatments. As such, it ought to aid one in accurately distinguishing between cases in which withholding/withdrawing treatment is justified by DDE and those in which it is not.

The aim of this paper is important for at least two reasons. First, there is an ongoing debate about the extent to which life-sustaining treatments are justifiably withheld/withdrawn. The debate began in the 1970’s, focusing on a few treatments, such as ventilator for comatose patients and cardiopulmonary resuscitation [30, Ch. 8]. Today, people continue to discuss the moral justifiability of such practices as, for example, removing a ventilator in patients with neuromuscular diseases [31], withholding a gastrostomy feeding tube in patients with dementia [32] and withholding dialysis in patients with kidney failure [33]. They want to know when and why these practices are justifiable. DDE is expected to provide criteria to distinguish between cases where withholding/withdrawing treatment is justified and those where it is not. Understanding exactly when each is the case can contribute to these on-going debates.

This paper’s aim is also important for another reason. The idea that active euthanasia and physician-assisted suicide are never morally justified, but that withholding/withdrawing life-sustaining treatment is justifiable in some cases, is still widely accepted internationally. True, in recent years, the number of countries and regions that have legalized active euthanasia and assisted suicide has increased rapidly. Nonetheless, they remain in the minority. On the other hand, withholding/withdrawing life-sustaining treatment is widely practiced throughout the world, and the idea that it can be ethically justified in some cases is almost universally accepted.

An intuitive way to explain the moral difference between active euthanasia and assisted suicide, on the one hand, and the withholding/withdrawing of life-sustaining treatment, on the other, is to say that the former is an action while the latter is an omission. However, this explanation has been sharply criticized as employing a distinction that is either conceptually confusing [19, Ch. 6] or morally insignificant [34–36]. Many who have argued that withholding/withdrawing treatment is justified only when the patient’s death is not intended have done so in response to these criticisms. According to them, the difference lies in the fact that the patient's death is always intended in active euthanasia and assisted suicide, but this is not so when a treatment is withheld/withdrawn [1, 2, 5, pp. 162–167; 8, pp. 71–80; 11]. Thus, an accurate understanding of how DDE applies to withholding/withdrawing treatment should also make an important contribution to assessing the medical policy in many countries and regions that currently prohibit euthanasia and assisted suicide.

The remainder of this paper proceeds as follows. The second section prepares my criticism of Brody and Sulmasy by looking at two ideas. One is that the treatment itself must be burdensome if it can be said that the patient’s death is not intended when a life-sustaining treatment is withheld/withdrawn. This is emphasized in the arguments of both Brody and Sulmasy, so I will explain why it is true. The other is that DDE requires the treatment burden to be grave enough so that the goodness of avoiding it compensates for the badness of shortening life. This is a requirement which follows from DDE’s fourth condition (that the act’s good effect is proportionately greater than its bad effect) as applied to withholding/withdrawing treatment and which Sulmasy claims is rarely met. Before seeing Sulmasy’s reasons for so claiming, I will explore the implications of this requirement to various concrete cases of withholding/withdrawing treatment. My discussion of this section must show that, contrary to Brody and Sulmasy's assertions, there seem to be numerous cases of withholding/withdrawing treatment that satisfy all the conditions of DDE, including the fourth. Then, in the third section, I will try to eliminate misconceptions contained in the arguments of Brody and Sulmasy.

## Applying DDE to withholding/withdrawing treatment

### The treatment must be burdensome

To be able to say that the patient’s death is not intended when a life-sustaining treatment is withheld/withdrawn, the treatment itself must be burdensome; that is, treatment must impose some substantial inconvenience, including physical, psychological, and economic burdens, on the patient, family, and/or society. This has been maintained both by those appealing to DDE to justify withholding/withdrawing treatment [1, p. 123; 4, p. 418; 5, p. 164] and Brody and Sulmasy [25, p. 98; 27, p. 125; 28, p. 441]. To see why this must be so, consider the following cases:

**Case 1: Blood transfusion**

A victim with injuries to both legs from a major car accident was brought to the emergency room. The doctor explained that she needed a blood transfusion to survive. She was fully conscious, understood that she would need a wheelchair to live the rest of her life if she survived, and refused the transfusion.

**Case 2: Antibiotics**

An infant’s parents became tired of parenting and abusive. When the infant developed a serious case of pneumonia, the doctor prescribed antibiotics. However, the parents did not give the medication to the infant.

These cases are not justifiable under DDE. This is because the treatment in these cases causes no substantial burdens or inconveniences to the patient, family, or society. For both blood transfusions and antibiotics, there is minimal to no discomfort. These treatments are also inexpensive.

In general, it is reasonable to assume that when one deliberately withholds/withdraws a life-prolonging treatment, one is trying to avoid some substantial inconveniences. It is important to distinguish between two types of inconveniences that one can avoid by doing so: those caused by the treatment itself and others. The latter usually stems from the illness or injury for which the patient needs the treatment, but this is not necessarily so. For example, in **Case 1**, the patient can avoid living in a wheelchair. This is an inconvenience stemming from the injury. In **Case 2**, the parents can avoid taking care of their baby. This is an inconvenience unrelated to the illness or injury.

In both **Cases 1** and **2**, the treatment itself does not cause substantial inconvenience. Accordingly, the agents in these cases cannot claim that their intention in forgoing treatment is to avoid the burden of the treatment itself. But this seems to mean that they cannot plausibly deny their intention to shorten life. Thus, these cases violate DDE’s second condition (that the agent ought not to intend the bad consequence of their act).

These conclusions may be objected to. It may be objected that, for example, the patient in **Case 1** may have only wanted to avoid living with a wheelchair and had no intention of shortening her life. Similarly, it might be thought that the parents in **Case 2** did not want to take care of the baby but had no intention to kill the baby. In fact, however, there are two reasons why these objections do not help to justify the cases.

First, even if these objections are correct, **Cases 1** and **2** still violate DDE’s third condition (that the act’s bad consequence ought not be the means to achieve its good consequence). In **Case 1**, the patient can avoid the burden of living in a wheelchair (assuming that it is a burden) only by eliminating herself. That is, if she refuses transfusion, then she will not need to use a wheelchair, but that is only because she will not be there to use it. In this sense, bringing about of her own death is a causal means necessary to achieve the good end. **Case 2** is similar. In the scenario, the parents can avoid the burden of taking care of their child only by eliminating the child.

Second, the objections could not be true in fact. DDE’s third condition is normally understood to have the function of making sure that the bad consequence of the act is not intended by the agent. For it seems undeniable that, as a matter of general fact, when one acts for an end, one cannot help but intend the consequence that one brings about oneself and that one knows to be a necessary means to achieve the end. Accordingly, when an act violates DDE’s third condition, it must also violate the second [13, pp. 29–30, 37; 38, pp. 91–92; 39, 40, p. 110]. In the previous paragraph, I showed that **Cases 1** and **2** violated the third condition; the patient’s death was a casual means to achieve the agents’ end. Accordingly, the cases also violate DDE’s second condition; it cannot be plausibly denied that the agents in these cases intended the patient’s death.

Two further comments are in order. First, as noted above, it is understood that active euthanasia and assisted suicide are never justified by DDE. The reason for this is also the point just made. In active euthanasia and assisted suicide, the physician either administers or prescribes a lethal drug to a suffering patient. One might say that the physician is only trying to relieve the patient from suffering and does not intend the patient's death. However, this cannot be true. For, in what they do, the physician can relieve the patient from suffering only by eliminating the patient. Their act thus violates DDE’s third condition. Additionally, given the account I explained in the paragraph immediately above, it must be said that the physician also intends the patient’s death as a means to their end. Their act thus violates DDE’s second condition as well [13, p. 109].

The last comment. In this section, I have explained that when one withholds/withdraws a treatment that is necessary to sustain a patient’s life but is not burdensome, it must be understood that the patient’s death is intended. However, even if the treatment is burdensome, it does not necessarily mean that the patient's death is not intended. In that case, the treatment burden may or may not be the only thing that the agent intends to avoid. For example, the agent may also aim to avoid the burdens stemming from the illness, in which case it must be understood that the patient’s death is also intended as a means to this end, or the patient’s death itself may be the agent’s end. In these cases, DDE determines that withholding/withdrawing treatment is not justifiable.^7^ I will come back to this point later in the third section.

### The treatment burden must be grave enough to compare the badness of shortening life

Above I’ve shown that the treatment itself must be burdensome if it can be understood that the patient’s death is not intended when the treatment is withheld/withdrawn. For the withholding/withdrawing of treatment to be justified by DDE, however, this is not enough. It must also be the case that the treatment burden is so great that the good of avoiding it compensates for the bad of shortening the patient’s life. Consider the following case:

**Case 3: Diabetic amputation**

Patient has worsening diabetes. His toes were necrotic, a condition that would be life-threatening only if not amputated. However, the patient refuses to undergo amputation.

Amputation is a physically and psychologically taxing procedure. Many patients hesitate to undergo this surgery when it is first proposed. In the present case, however, it seems clear that the advantages of avoiding this burden are not great enough to compensate for the disadvantages of giving up life. If so, forgoing amputation in this case is not justified by DDE for it violates the doctrine’s fourth condition.

Comparing the treatment burden with the goodness of maintaining life requires a value judgment. While I believe reasonable people would agree that the treatment burden in **Case 3** is (though not insignificant) proportionately too small to allow for the shortening of life, their judgments may differ over other cases. I will list some of those cases later. But before then, consider cases where their judgments would agree in the opposite direction, i.e., in that the treatment burden is large enough to allow for the badness of shortening life.

Chemotherapy is frequently mentioned in prior studies as an example of a treatment that is justifiably forgone [1, pp. 123–124; 5, p. 161]. Chemotherapy is a treatment that can impose a large burden on patients. Common side effects include generalized pain, loss of strength, hair loss, and mouth ulcers. Simultaneously, the life-sustaining effects of the drugs are often not promising, especially for patients with advanced cancer. In these cases, withholding the treatment may mean that the patient can spend the remaining time more meaningfully while giving up a small chance to sustain life longer. It would then seem plausible that the benefit can be large enough to compensate for the disadvantage. For example, consider the following case:

**Case 4: Chemotherapy**

A patient with advanced cancer is told that there is a 20–30% chance that chemotherapy will keep her alive for five months to a year. If she does not receive the treatment, she is very likely to die sooner (probably in a couple of months). The patient refused the treatment to avoid the side effects.

This case is justifiable by DDE. First, saying no to the treatment (the act itself) is morally indifferent.^8^ Second, it can be understood that the patient only intends to avoid the side effect but not to shorten her life. Third, shortening of life is not the means to avoid the side effect. Forth, the benefit of avoiding the side effect is considered proportionately serious enough to accept the small chance of dying sooner.

Note that in **Case 4**, the treatment has only a small chance to improve the patient’s condition and would not improve much if it did. However, this does not have to be so for DDE to justify the withholding/withdrawing treatment. Even if the treatment is certain to improve the patient's condition, withholding/withdrawing it can be justified if giving up the improvement is less bad than the inconvenience that the treatment would impose.^9^ Consider another case to see this point.

Dialysis and other renal replacement therapies have long been the standard of care for patients with end-stage kidney disease. However, in recent years, several studies have been published reporting that the initiation of dialysis does not necessarily promote the best interests of patients [33, 41, 42]. For example, W. R. Verberne and colleagues studied patients with end-stage kidney disease who were 70 years old or older. Patients who initiated dialysis were compared with those who withheld it. The median survival from the time of considering starting dialysis was 4.3 and 2.4 years, respectively, nearly twice as long for the former than for the latter. However, the burden of treatment in terms of hospital visits, hospitalization, and costs was significantly greater in the group that initiated dialysis. Additionally, the difference in life expectancy tended to be smaller the older the patient was [33]. Given these results, it seems reasonable to believe that withholding of dialysis can satisfy all the DDE’s conditions in some cases; for example:

**Case 5: Dialysis**

The patient is 73 years old and has end-stage kidney disease. The physician informed him that if he forgoes dialysis, he would likely die in about two years, but that there was a significant chance that starting dialysis would extend his prognosis by a couple of more years or so. While also realizing that living two years longer would be of great value, the patient decided against dialysis because he wanted to avoid the burden of hospital visits.

This is not to say that DDE always justifies withholding chemotherapy or dialysis. First, these treatments can be much more effective in prolonging life. In early-stage cancers, chemotherapy can often be expected to induce a complete remission. For younger patients with kidney failure, dialysis may extend their prognosis by decades. In these cases, it must be clear that avoiding the treatment burden is proportionately too small to compensate for the shortening of life. Withholding treatment would then violate DDE’s fourth condition.^10^

Conversely, although I stated above that DDE prohibits forgoing limb amputation in some cases (**Case 3**), it may also justify it in other cases. Amputation is not always very effective in preserving life. Forgoing it may be justified when the burden is deemed onerous enough in proportion to the life preserved. (However, I will come back to discuss amputation more in section three. There is still an issue that must be resolved to determine whether forgoing amputation is justified by DDE.)

The withholding/withdrawing of various other life-sustaining treatments can be considered in the same manner. For example, consider cancer surgery, gastrostomy feeding tube for dementia patients, antimicrobial therapy for elderly pneumonia patients, and various organ transplant procedures. All these procedures can cause onerous burdens in the form of pain, discomfort, loss of strength, and others. At the same time, depending on the patient's condition, their life-sustaining effects may not be great. When the burden is considered large enough to allow for giving up sustaining life, then withholding/withdrawing of these treatments can be justified by DDE.

As above noted, the determination of whether DDE justifies withholding/withdrawing treatment requires a comparison of values, and people's judgments on this point may not coincide. Cases where the judgments are particularly likely to diverge are those in which a treatment would be expected to prolong life for an extended period, such as the following.

**Case 6: Blood transfusion and Jehovah's Witness**

A Jehovah's Witness believer has been in a car accident. Although she lost a life-threatening amount of blood, a complete recovery is possible if she receives a blood transfusion. However, she refused the treatment because it is against her faith.

**Case 7: Ventilator for advanced motor neuron disease**

A patient with amyotrophic lateral sclerosis (ALS) requires a tracheotomy to sustain life. Despite the fact that a tracheotomy and ventilator can prolong life by years, he refused the procedure because he did not want to lose the ability to speak with his own voice.

**Case 8: Treatment of burns (Dax Cowart's Case)**

Mr. Cowart was involved in a gas explosion and suffered severe burns all over his body. The treatment was extremely painful and had to be repeated over a long period of time for him to survive. He was told that he would be able to return to his daily activities after treatment, but he did not agree to continue his treatment [43].

For these cases, difficult judgment is required to compare the value of long-term survival with the badness of living against one's faith, the physical and psychological burden of a tracheotomy, and the great pain, respectively.

Accordingly, one should expect disagreement over whether withholding/withdrawing treatment is justified by DDE in these cases. One can also imagine that there are many cases in which people do agree that it is not justified by DDE (as in **Cases 1** through** 3**). Nevertheless, the discussion in this section should have revealed that DDE also justifies it in many cases. The next section will look at some objections that have been presented to this conclusion and show that they are mistaken.

## Eliminating misconceptions

### Is the patient’s death rarely unintended?

Baruch Brody rejects the view that “decisions to withhold or withdraw [life-sustaining] therapy are justified because the death of the patient is not intended either as the end of the decision or as the means to attain the end” [25, p. 99]. Relatedly, he also dismisses the idea that euthanasia is morally different from withholding/withdrawing life-sustaining treatment because the patient’s death is always intended only in the former but not in the latter [25, pp. 98–99].^11^ He rejects these ideas because he believes that the patient's death is rarely unintended as a matter of fact when life-sustaining treatment is withheld/withdrawn. Here is how he expounds:

On their account, the end is avoiding the burdens of the treatment, and the death of the patient is not even the means to attain that end […]. That account is not consonant with much of my clinical experience […]. In many cases of withholding or withdrawing therapy, it is the continued existence of the patient in the condition in which they are in [sic] which is found burdensome, not the treatment itself. “Mama wouldn’t have wanted to live this way” is the common refrain, and withholding or withdrawing of therapy are undertaken in response to that refrain. The death may be the intended end for which the decision is made, or, more plausibly, the intended means to avoid the continued suffering and indignity of living that way. It is certainly not a mere foreseen side effect [25, p. 99].

Brody's rationale for thinking that the patient's death is not "a mere foreseen side effect" is that what people actually find burdensome and want to avoid when withholding/withdrawing treatment is "the continued existence of the patient in the condition in which they are” and not “the treatment itself.” He also supports this argument with his observation that the patient’s family members often say things like "Mama [= the patient] wouldn't have wanted to live this way" when withholding/withdrawing treatment.

There is a fallacy in this argument. Assume, following Brody, that it is often "the continued existence of the patient in the condition in which they are" that people see as a burden, and intend to avoid, when treatment is withheld/withdrawn. In fact, this seems likely. However, that does not necessarily mean that people intend for the patient to die. I will explain why shortly. Before that, however, let me say a few words to ensure an accurate understanding of his claim.

As above said, Brody rejects the view that withholding/withdrawing treatment is “justified because the death of the patient is not intended.” In the quoted passage, however, he merely asserts that his clinical experience shows that the patient’s death is intended in “many cases of withholding or withdrawing therapy”; since he does not state that death is always intended when a treatment is withheld/withdrawn, his argument cannot be that the view is wrong because its requirement for justification of withholding/withdrawing treatment is never satisfied. I take him to be arguing that the view is wrong because it justifies too few cases of withholding/withdrawing treatment. To put it more precisely, he must be arguing that the view is wrong because there are many cases of withholding/withdrawing treatment that are clearly justifiable but are prohibited by the view. I aim to show that this is mistaken. As I argue, the view he is criticizing, if properly understood, does not prohibit the cases that he believes it prohibits. To see why, consider the following points.

Brody distinguishes between "the continued existence of the patient in the condition in which they are" and "the treatment itself." He argues that when a treatment is withheld/withdrawn, it is often the former that is seen as a burden, not the latter. However, in the first place, these two burdens cannot be distinguished as sharply as Brody thinks. For example, for a patient undergoing chemotherapy, the fact that they are undergoing chemotherapy is part of the condition in which they are. Hence, if chemotherapy is a burden for them, then continuing to live with it is also a burden. Simply put, Brody sees two kinds of burdens here: the burden of staying alive in the situation the patient is in (*c*) and the treatment burden (*d*). However, these burdens are either the same or in a relationship in which *c* is partially or entirely caused by *d*.

The same is true with the family’s complaint that “my mother would not have wanted to live this way.” It is still possible that the family sees the treatment that is sustaining the mother’s life as a burden to the mother. For it may be the very treatment that is forcing the mother to live that way.^12^

To be sure, there are cases in which the burden of staying alive is unrelated to the burden of treatment. This is when the treatment causes no burden. Recall **Cases 1** (blood transfusion) and **2** (antibiotics). In these cases, the treatment itself imposes no substantial burden on the patient, and any burdens that can be avoided by foregoing treatment can only be avoided via eliminating the patient's existence; that is, it cannot be denied that the patient’s death is intended. Hence, these cases are not justified by DDE. Brody’s reasoning is valid as far as it concerns these cases. However, not all cases are like them. Many treatments that help to sustain life impose a grave burden on the patient.

Surely, even when the treatment itself is burdensome, that only ensures that the patient’s death is not necessarily intended; it is still possible that death is intended (see my comment at the end of the first subsection of the second section). In these cases, the burden of staying alive may be caused both by the treatment and the illness; if one’s aim in withholding/withdrawing treatment is to avoid the part of the burden that is caused by the illness, then, one can achieve this aim only by eliminating the patient’s existence. However, there is also a reason to believe that such cases are not very common in fact. Notice that when a treatment is said to be necessary to sustain or prolong life, in most cases, it is not certain that the patient will die immediately once the treatment is withheld/withdrawn. Rather, usually the patient will continue to live for some time, free from the burden of the treatment. For example, when chemotherapy is considered necessary to prolong life but is decided to be forgone, the patient does not die immediately with the decision. They continue to live for months or longer, free from its side effects. It seems plausible that many patients forgo the treatment because they think those months are precious. Thus, especially in these cases, there is a reason to believe that many patients do not intend to die early but only intend to enjoy the time free from the treatment burden.^13^

In other words, ceasing to live in a difficult condition is different from ceasing to live per se, and it is possible to intend the former without intending the latter. To be sure, when a treatment is truly necessary to prolong life, the patient will die sooner, if not immediately, if the treatment is withheld/withdrawn than if it is not. In this sense, one cannot avoid the burden of treatment without causing an earlier death. However, this does not mean that earlier death is also intended when the treatment is withheld/withdrawn. In the terms commonly used in the DDE literature, an earlier death in this case is merely necessarily concomitant to, but not causally necessary for, the agent’s goal (i.e., to avoid burden). What a rational agent cannot help but intend is what is causally necessary to achieve their goal. What is merely necessarily concomitant to their goal can be a mere side-effect of their act [45, p. 353].

To summarize, people often explain their reason for refusing treatment by saying that they do not want to live the way they live. However, this does not necessarily mean that they intend to die early. Rather, contra Brody, it is plausible to assume that in many cases, they only intend to avoid the burden of the treatment, and an earlier death is a mere side effect.

### Does treatment burden rarely compare the badness of shortening life?

Daniel P. Sulmasy maintains that life-sustaining treatments are sometimes rightly deemed extraordinary and hence justifiably withheld/withdrawn. However, he also contends that the claim that DDE provides justification for such withholding/withdrawing is an instance of misapplication of the doctrine. As he explains, this is because withholding/withdrawing of life-prolonging treatment seldom satisfies DDE’s fourth condition (that the good effect of the act is proportionately serious enough to permit its bad effect). While the point is reiterated in some of his articles [26, p. 547; 27, 28, p. 441], it is elaborated most carefully in his 2005 paper, in which he argues as follows:

Under RDE [= rule of double effect], one may only consider the effects that flow directly from one’s action. … The good effect that results directly from the discontinuation of the treatment is the cessation of pain or other discomfort caused by the treatment itself. The bad effect is death. This sets an extremely high standard for determining that the discontinuation of life-sustaining treatment is morally licit. It would seem that one could only consider a treatment “extraordinary” if the treatment itself caused suffering proportionately graver than the evil of shortening life [27, p. 125].

When applied to withholding/withdrawing treatment, DDE’s fourth condition requires that the treatment burden is worse than the shortening of life. Here, Sulmasy is saying that this “sets an extremely high standard.” I have already shown above (in the second section) why I believe this is not so by applying the requirement to various concrete cases. True, a difficult value judgment is sometimes required to determine whether the treatment burden is worse than shortening life. Still, treatments such as anticancer drug administration, tumor removal, dialysis, amputation, and organ transplantation can all impose a grave burden on the patient. One can well imagine that these burdens may reasonably be judged to be proportionately graver than the badness of giving up improvements in the condition, especially when the expected effects of the treatment are less than ideal. Thus, it seems obvious that the requirement is satisfied in many cases of withholding/withdrawing treatment.

Why does Sulmasy believe otherwise? In the quote above, he maintains that only “the effects that flow directly from one’s action” can be taken into consideration when deciding whether the action is justified by DDE. In the case of withholding/withdrawing treatment, as he explains, this means that one can only consider “cessation of pain and other discomforts caused by the treatment itself” as “the good effect” of the act (= withholding/withdrawing treatment). Surely, the disease or injury for which the patient needs treatment may also cause discomforts, and one can also stop these discomforts by withholding/withdrawing the treatment. However, as Sulmasy contends, this latter effect should not be considered because it does not flow directly from the act, but is brought about via elimination of the patient’s existence.

Sulmasy elaborates this point by contrasting DDE with another traditional idea in Catholic moral theology. According to him, already in the seventeenth century, Catholic theologians classified life-sustaining treatments into the ordinary and extraordinary and argued that only the latter was permissibly withheld/withdrawn. Sulmasy calls this the O/E distinction. Now, however, while DDE and O/E distinction both belong to Catholic tradition, they are historically unrelated to each other in the tradition and cannot be equated in terms of content. Importantly, as he understands, if DDE is applied to withholding/withdrawing treatment, it would generate very different judgments than O/E distinction. Sulmasy’s point is that this occurs because only DDE, but not O/E distinction, should ignore the burdens that illness imposes on the patient when judging whether a treatment is justifiably withheld/withdrawn. To be more specific, as he explains, the O/E distinction deems a treatment extraordinary when the sum of the treatment burden plus the burdens caused by the illness exceeds what one can reasonably endure in trying to fulfill the moral obligation to preserve life [27, p. 123]. In his own words, “the suffering associated with the condition itself, independent of the treatment, is part of the burden to be considered in judging whether a life-sustaining treatment is ordinary or extraordinary” [27, p. 128]. In contrast, under DDE, “one could only consider a treatment ‘extraordinary’ [= non-obligatory] if the treatment itself caused suffering proportionately graver than the evil of shortening life” [27, p. 125] (in the direct quote above). “In this sense,” continues Sulmasy, “the proportionality condition of the O/E distinction differs dramatically from the proportionality condition [= the fourth condition] of the RDE [= rule of double effect]” [27, p. 128]. His claim is that this makes it very difficult to fulfill DDE’s fourth condition by withholding/withdrawing treatment.

Surely, then, the next question to be asked is why Sulmasy holds that one can consider only those effects that “directly flow from” the act, or the termination of only those burdens caused by the treatment itself, when determining whether withholding/withdrawing treatment satisfy DDE’s fourth condition. He provides what appears to be his answer to this question in a later section of the same article. There, it is argued that if one intends the termination of other kinds of burdens when withholding/withdrawing treatment, then one must also intend the patient’s death, but this is prohibited by DDE. He argues this by reflecting on cases in which “the treatment is not causing much suffering in itself […]” [27, p. 131]. In such cases, if one withholds/withdraws the treatment, the intention can only be to relieve the suffering that is caused by the disease. He maintains that DDE does not justify such withholding/withdrawing because “[o]ne could only bring about the intended good by means of the death of the patient, violating the 3rd condition of the classical RDE. […] One cannot reasonably claim to intend to do p, knowing that it brings about q as the means by which r comes about, intending r but not intending q” [27, p. 131]. This is precisely the point I made above in the second section.

To put it simply, then, Sulmasy’s reasoning seems the following. He observes (correctly) that DDE allows one only to aim at removing the burden that treatment itself brings when one withholds/withdraws treatment. For otherwise, one would violate DDE’s third condition (that the good effect ought not be caused by means of the bad effect). However, this has led him to believe that the badness of the burdens caused by illness should not be considered at all when DDE is applied to withholding/withdrawing treatment. Sulmasy also repeats this argument in another paper [28].^14^

I have two comments on this argument. First, if it is true, DDE would hardly seem like a reasonable standard for assessing the morality of withholding/withdrawing treatment. For it seems undeniable that whether patients should be allowed to die by refusing treatments depends, at least in part, on how much they suffer from the illness.

Second, however, there is a flaw in Sulmasy’s argument. Even with DDE, to determine whether withholding/withdrawing treatment is justified, one must consider how strongly the disease is afflicting the patient, in addition to the burden that the treatment itself imposes on the patient. Here, it is important to distinguish two things. One is to aim at removing burdens caused by the illness (*e*), and the other is to assess the magnitude of burdens caused by the illness (*f*). When applying DDE to withholding/withdrawing treatment, *e* is prohibited, but *f* is required. According to DDE, a person's goal in withholding/withdrawing treatment must be to remove the burden caused by the treatment itself. So, *e* is prohibited. At the same time, however, DDE also asks one to confirm that the removal of the burden caused by the treatment itself is so good that it compensates for the badness of the shortening of the patient’s life. To confirm this, one needs to assess how bad it is to shorten the patient's life. It is here that one is called upon to do *f*. For to assess the magnitude of the burdens of illness is mostly, if not entirely, a matter of evaluating the extent to which the illness has affected the patient's quality of life. And it must be thought that the more the illness has impaired their quality of life, the less bad it is to shorten their life. Note also that there is nothing inconsistent or irrational in doing *f* without doing *e* at the same time.

To illustrate my point, let me discuss a case that Sulmasy provides in his article. He believes the case does not fulfill DDE’s fourth condition. It is fiction concerning a male patient with a particular neurological disease. The disease renders the patient docile and unable to make decisions, and causes severe facial pain for which palliative measures are ineffective. It also renders the patient unable to breathe spontaneously, making him dependent on a ventilator to survive [27, p. 120]. In Sulmasy's view, while his family's decision to remove the ventilator is morally justifiable, no rationale for it is given by DDE. This is because the burden caused by the ventilator itself is relatively small. That is, “The ventilator is not causing pain” and “The tracheostomy is not uncomfortable” [27, p. 122]. Hence, for him, “the discomforts of the ventilator itself pale” in comparison with the badness of shortening life. Accordingly, as Sulmasy concludes, “it is implausible … to suggest that she [= the patient's wife] would have a proportionate reason for discontinuing the ventilator if her intention was to relieve [the patient] of the discomfort caused by the ventilator” [27, pp. 121–122].

The correctness of Sulmasy’s assessment is not obvious. Assume with him that the burdens caused by the ventilator are not very large. Even still, the case may well satisfy DDE’s fourth condition. For it is also plausible that the patient's quality of life is significantly diminished because of the illness. The patient is incapable of mental activity due to diminished consciousness and suffers from severe pain. A shortening of such a life may not seem like a very bad thing. That is, even if the goodness of avoiding the treatment burden is not very large, it may still suffice to compensate for the badness of shortening life.

Accordingly, the patient’s family may reasonably believe that turning off the ventilator satisfies DDE’s fourth condition. Surely, their belief as such would be based on an assessment in which the burdens caused by the illness are also taken into consideration. Importantly, however, this does not necessarily mean that they would intend to shorten the patient’s life by turning off the ventilator. Their thought would be that the illness has rendered the quality of the patient’s life so low that the shortening of that life is acceptable if the burden of the treatment can be removed. Even then, it is clearly possible that the family only intends to remove the treatment burden, and the shortening of life is understood as a mere side-effect.

### Does DDE always prohibit amputation?

I now move to a final misconception. In the second section, I stated that the forgoing amputation may violate DDE’s fourth condition in some cases, but can be justified by the doctrine in other cases. However, Sulmasy also denies this. In amputation, the removal of a limb preserves the patient's health and life. Obviously, the removal of a limb is a bad thing, and the preservation of health and life is a good thing. Accordingly, he argues that in amputation, a bad outcome is the means to cause a good outcome, thus violating the third condition of DDE [29, p. 89].^15^ If his argument is correct, then DDE would not sometimes justify forgoing amputation; rather, it would always demand forgoing amputation.

Following the same reasoning, DDE would always prohibit even more routine medical practices. Consider palpation: the physician touches the patient's body to identify the affected area, saying, ‘Let me know if you have any pain.’^16^ Here, the occurrence of pain is a causally necessary means of identifying the affected area. Obviously, however, pain is a bad thing, and being able to identify the affected area is a good thing.

It is clear that amputation and palpation are justified in some cases. Therefore, if DDE were to prohibit these practices altogether, it must mean that DDE is an erroneous doctrine.^17^

However, it is a mistake to think that DDE should always prohibit amputation or palpation. DDE is not a principle that generally prohibits one’s intending to bring about bad outcomes as a means to a good end. As noted in the first section, behind traditional DDE is the theory of moral absolutism. This theory maintains that some bad consequences are such that one should never intend to bring them about; for example, the death of an innocent person. Call this type of bad outcome especially bad. However, absolutism does not state that one should never intend to bring about any bad consequences. Thus, for example, it may regard the loss of a limb or pain as something that, although bad, one may sometimes intend to bring about. Call this type of bad outcome merely bad. Now, the traditional DDE applies only to those acts that cause especially bad consequences. Its purpose is to provide conditions under which an act can be justified despite causing such bad consequences [14, p. 58; 15, p. 476; 47].

In other words, traditional DDE does not apply to actions that bring about merely bad consequences. Again, it is possible to consider the loss of a limb or pain to be a merely bad outcome.^18^ In that case, DDE would not apply to acts that are only foreseen to result in a loss of a limb or moderate pain. This is why it is possible to think that DDE does not prohibit amputation or palpation.

My argument may be disputed. First, traditionally, proponents of DDE often seem to have understood that maiming the innocents, like killing them, should never be intended [13, p. xv, n4]. This suggests that any act of severing a limb, including amputation, must satisfy the conditions of DDE to be justifiable. If this is so, my argument deviates from the traditional conception of the doctrine. Relatedly, second, one may think that it is ad hoc to maintain that the doctrine applies to the causing of death but does not apply to the causing of other very great harms, like maiming. Further, there are many treatments with serious but non-fatal side effects. My argument entails that DDE has no role in explaining why these treatments are often acceptable. However, one might also think that DDE ought to explain this.^19^

While important, these objections are far from conclusive. Two comments are in order. The main thrust of these objections is that my argument makes DDE more limited in its scope of application than is often believed. This may seem to be a drawback to my argument. First, however, it does not follow that my argument is flawed unless it is also shown that DDE ought to account for a broad range of issues. Second, drawing the line as I have done is not entirely ad hoc. Rather, it seems plausible to consider the destruction of life to be on a higher order of badness than the destruction of a body part. My argument is not to deny that destruction of a body part can be a very bad thing. Nor do I try to deny that causing the destruction of a body part by intending it can be worse than causing it without intending it. I claim that only the destruction of a body part, but not the destruction of life, is justifiably intended when done for a proportionately greater good. I believe that this claim would be endorsed by many people; at least, it must be shared widely by those who support a total ban on euthanasia but do not oppose amputation.

## Conclusions

This paper examined the view that withholding/withdrawing life-sustaining treatment is justified when it is intended to avoid the burdens caused by the treatment itself but not to shorten the patient’s life. Brody and Sulmasy independently criticized this view. I have argued that their criticisms are based on a misunderstanding. According to Brody, those who refuse life-sustaining treatment because they find their present way of life intolerable have the intention of shortening their own lives. Sulmasy maintains that those who decide to withhold/withdraw treatment in consideration of the amount of the suffering caused by the illness are aiming to avoid this suffering by eliminating themselves. I have shown that these claims are simply wrong in many cases. I have also eliminated a further misconception that DDE should always prohibit such commonplace medical procedures as amputation or palpation.

Once these misconceptions are dispelled, it should become clear that DDE can justify many cases of withholding/withdrawing treatment and distinguish them from unjustified cases in a consistent and intuitively appealing manner. DDE is a promising criterion for determining the range of treatments that are justifiably withheld/withdrawn. Also, many countries and regions currently prohibit euthanasia and physician-assisted suicide outright, while partially permitting withholding/withdrawing life-prolonging treatment. DDE provides a rationale for believing that such policies are appropriate.

## Footnotes

1 Hereafter, by “treatment,” I mean a treatment that is necessary to sustain a patient’s life unless otherwise noted.

2 DDE is sometimes formulated as consisting of three conditions rather than four [13, p. 36]. There is a legitimate reason for this. As will be shown in the second section, it is evident that when an act violates the third condition, it also violates the second condition. This indicates that the third condition can be omitted.

3 Today, DDE is sometimes understood in a form divorced from absolutism. The doctrine is then formulated more simply as follows: It is easier to justify an act when its bad effect is not intended than when it is intended, other things being equal [17, 18]. The DDE thus formulated is called the secular, non-absolutist version. Suppose the secular version is correct. Then, even assuming that the death of the patient is always intended for active euthanasia or assisted suicide, that does not necessarily mean that DDE never justifies these acts. Compare this to how DDE in its traditional form applies to the same acts (which I explain in the text below). My discussion below will focus on the traditional DDE.

4 In this respect, my project to defend the idea that DDE provides a moral justification for withholding/withdrawing treatment is incomplete. For a critique of DDE itself, see [19, Ch. 11; 20, 21]. For its defense, see [13, Ch. 3&4; 22, 23].

5 Virtually all prior studies endorse the general statements. For especially clear statements, see, for example, [4, p. 418; 9, pp. 162–163].

6 This does not mean that DDE always justifies withholding/withdrawing treatment if all three requirements specified in the present paragraph are satisfied. Indeed, there seem to be cases where withholding/withdrawing treatment is clearly unjustifiable even if it satisfies the three requirements, for example, when the patient autonomously decides for the treatment. It is not necessary to assume that DDE should justify such cases. In fact, it is plausible that withholding/withdrawing treatment against the patient's autonomous choice is an act that is bad in itself. This indicates that the case is ruled out by DDE’s first condition (I am grateful to one of the anonymous reviewers for presenting this reasoning). Alternatively, it may be thought that the goodness of avoiding the treatment burden is never proportionately serious enough to allow for a violation of patient autonomy. If so, the case is prohibited by DDE’s fourth condition (Allison McIntyre suggests this reasoning [21, p. 223]). Note also that some philosophers maintain that DDE does not provide a sufficient condition for an act to be justifiable. For them, an act may satisfy all four conditions of DDE simultaneously and yet be impermissible because it violates other important moral principles [24, p. 21]. Following this account, one may argue that withholding/withdrawing treatment against the patient’s wish is unjustifiable even if it satisfies all of DDE’s four conditions because it violates the principle that the autonomous choice of the patient ought to be respected.

7 Consider, for example, parents who refuse surgery to correct a bowel obstruction in a newborn with Down syndrome. Many argue that this is not justified by DDE [1, 2, 5, pp. 161–162]. In this case, the treatment burden is small (i.e., the surgery is simple and risk-free), yet it may not seem to be entirely absent. Even so, it seems obvious to many that the parents’ intention is to avoid raising a child with Down syndrome.

8 I believe that this is uncontroversial. DDE’s first condition requires that an act in itself be good or morally indifferent. The premise is that certain acts are intrinsically bad, i.e., bad independently of their consequences, and are therefore excluded even before proceeding to the stage of examining whether they satisfy the other three conditions (where the consequences first become an issue) [8, p. 91; 13, pp. 27–28; 16, p. 465]. While murder is always cited as an example of an intrinsically bad act, it can be debated what other acts are included in the list of such acts and whether the theological explanations traditionally given to justify the list have validity [16, p. 465]. No matter how one understands the list, however, it seems clear that it does not include the act of refusing treatment, as conceived independently of its consequences, i.e., shortening of life. Sulmasy also states that “the first condition of RDE [= rule of double effect] is easily satisfied” in the context of applying DDE to the removal of a ventilator [27, p. 121].

9 The point deserves emphasis. It is very common among those who maintain that DDE provides justification for withholding/withdrawing life-sustaining treatment to emphasize that the treatment has a very small chance of improving the patient’s condition when they describe justified cases. For example, according to Bonnie Steinbock, termination of life-sustaining treatment is not identified with “intentional termination of life” when “continued treatment *has little chance of improving the patient’s condition* and brings greater discomfort than relief” [1, p. 123, emphasis added]. Thomas Sullivan defines “extra-ordinary means of preserving life” as “all those medications, treatments and operations which cannot be obtained without excessive expense, pain, or other inconvenience, or which, if used, *would not offer a reasonable hope of benefit*” [2, p. 135, emphasis added]. Likewise, in applying DDE to withholding/withdrawing treatments, Robert Campbell and Dianne Collison write that "Declining to adopt measures with *little or no prospect of success* and which additionally involve risk, hardship or disproportionate pain for the patient, is perfectly justifiable” [5, p.164, emphasis added].

10 Note that it is also possible that DDE is not applicable to the withholding of these treatments. According to the same study by Verberne and colleagues, the difference in life expectancy between the group that initiated dialysis and the group that withheld it disappeared when the patients were over 80 years old or had multiple serious comorbidities [33]. Thus, one may reasonably believe that withholding the treatment for a given patient does not shorten life (in other words, that the treatment is futile) when the patient is very old or very sick. This means that while the act is likely justifiable, DDE does not provide a rationale for it. Recall that DDE only applies to acts that have at least one good and one bad foreseeable consequence (c.f. Introduction). The point of appealing to the doctrine is to show that even actions known to have bad consequences can sometimes be justified. If the act is known to have no bad consequence, there is no need to appeal to DDE.

11 The overall aim of Brody’s paper is to investigate “whether, from the moral perspective, the withholding or withdrawing of life-preserving therapy is significantly different than the administration of lethal drugs” [25, p. 91]. For this aim, he examines what he calls “the intending death account,” according to which “the decision [that foreseeably results in an earlier death of a patient] constitutes a decision to kill if the earlier death of the patient is intended as an end, or as a means to attain an end, for which the decision is made, and constitutes a decision to let die if the earlier death of the patient is foreseen but not intended in either way” [25, p. 97]. Brody maintains that those who hold this account “usually claim” that withholding/withdrawing life-sustaining treatment is justified, when it is justified, because the death of the patient is not intended [25, p. 99]. It is this latter claim (call it *P* for convenience) that he is criticizing in the passage quoted in my text below. Brody also rejects the intending death account itself for a different reason: He refers to a survey conducted among Dutch physicians, according to which not prolonging life was their explicit goal in about half of the cases where treatment was withheld/withdrawn before the patient died [44]. As he explains, the treatments withheld/withdrawn in these cases presumably include “CPR, intubation, dialysis, blood products, antibiotics and so forth,” but this shows that the intending death account is false because decisions to withhold/withdraw these treatments are normally understood as “paradigms of decisions to let patients die, rather than as decisions to kill” [25, p. 98]. Suppose this argument is correct. Still, it does not show that *P* is false. For it may be the case that withholding/withdrawing these treatments is not killing but unjustified nonetheless. Also, regarding the Dutch study, it is worth noting that the results would likely be different if it had been conducted in other countries where euthanasia and assisted suicide are not legalized. In the Netherlands, at least legally, it is not unjust to bring about a patient’s death by intending it. So, Dutch physicians may resist less strongly than physicians in other countries to bring about a patient’s death by intending it. If so, physicians in other countries are less likely to intend to shorten life in withholding/withdrawing treatment when shortening life does not have to be intended and less likely to withhold/withdraw treatment when shortening life must be intended.

12 I understand the word “condition” in Brody’s passage (quoted above) in a broad sense, i.e., as referring to the overall condition the patient is in. Thus, I take him to be arguing, problematically, that most patients and families who withhold/withdraw treatment intend to avoid the burden of staying alive in the patient’s condition as such, but few of them intend to avoid treatment burden. The argument is problematic because these two burdens are often the same. Now, one might think that Brody is using the word more narrowly, i.e., to mean the pathological condition or disease state of the patient (I thank one of the reviewers for suggesting this reading). This reading would allow Brody to neatly distinguish between the burden of staying alive in the condition the patient is in and the treatment burden. His claim would then be that most patients and families intend to avoid the burdens caused by the disease, and few of them have the intention to avoid treatment burden. However, this claim must also be wrong if I am correct in what I say below in the text, i.e., that many patients do seem to intend to avoid the treatment burdens only and do not intend to die early when withholding/withdrawing treatment.

13 In some cases, one’s decision to withhold/withdraw a life-prolonging treatment causes the patient's immediate death; for example, when cardiopulmonary resuscitation is withheld or when one turns off the ventilator of a patient who is completely dependent on the device for life support. In these cases, clearly, one’s intention is not for the patient to enjoy the time free from the burdens of treatment. But even in these cases, it is still possible that one only intends to avoid placing the burden of the treatment on the patient and does not intend to let the patient die. This is also because the immediate death of the patient is not causally necessary to avoid the treatment burden even in these cases; it is merely necessarily concomitant to this goal. See my text below.

14 Here I take him to be arguing that the magnitude of the treatment burden ought not be considered in determining whether the good effect of withholding/withdrawing treatment is proportionately greater than its bad effect because doing so is prohibited by DDE’s third condition. The argument is expressed more clearly in his later article, in which he maintains: “In DER [= double effect reasoning], the benefits and burdens one weighs are those directly caused by one’s act. In considering the withdrawal of an extraordinary treatment, one also considers the burdens caused by the disease, not just the treatment itself. If limited to strict DER reasoning, very few treatments could be stopped because the only way the relief of suffering caused by the disease could be construed as an effect of stopping the treatment would be by the patient’s death, making death the intended means of achieving one’s good, which DER would prohibit. Lumping the withdrawal of extraordinary treatment under the broad label DER thus leads us to serious errors in thinking about care at the end of life” [28, p. 441].

15 Elizabeth Anscombe also recognizes this as a pressing problem for DDE [46, pp. 217–219]. See my footnote 18 below.

16 Allison McIntyre discusses essentially the same case [21, p. 228].

17 Sulmasy does not seem to understand this as a challenge for DDE. For him, DDE is only one among many principles of medical ethics, and amputation can be justified by other principles [29, p. 89]. Usually, however, it is understood that failure to satisfy any one of the conditions of the DDE is a sufficient condition for an act to be morally prohibited. It is logically inconsistent to embrace with DDE some other principles that justify an act that DDE prohibits. Also, Frances Kamm maintains that traditional DDE associated with absolutism is problematic in that it absolutely forbids intentionally causing even relatively minor harms such as "moderate pain” [17, p. 572]. As I will show below, however, absolutism need not be understood as Kamm understands it.

18 For example, Anscombe conceives DDE (or, more precisely, the part of DDE that absolutely prohibits seeking someone’s death either as an end or a means, which she calls the principle of side effect) as “particularly devised for the causing of death; causing of other harms is not covered by it” [46, p. 225]. As noted above (my footnote 15), Anscombe recognizes that amputation cases pose a pressing issue for DDE. However, I also understand her to be suggesting (by the passage I have just quoted) that amputation cases do not in fact undermine this doctrine, and that the reasons for this can be explained in the manner I describe in the text.

19 I am grateful to two of the anonymous reviewers for raising these points.
